# Evaluation of the branched-chain amino acid-to-tyrosine ratio prior to treatment as a prognostic predictor in patients with liver cirrhosis

**DOI:** 10.18632/oncotarget.18447

**Published:** 2017-06-12

**Authors:** Toru Ishikawa, Michitaka Imai, Masayoshi Ko, Hiroki Sato, Yujiro Nozawa, Tomoe Sano, Akito Iwanaga, Keiichi Seki, Terasu Honma, Toshiaki Yoshida

**Affiliations:** ^1^ Department of Gastroenterology and Hepatology, Saiseikai Niigata Daini Hospital, Niigata 950-1104, Japan

**Keywords:** liver cirrhosis, branched-chain amino acid, branched-chain amino acid-to-tyrosine ratio, event-free survival

## Abstract

This study evaluated whether the branched-chain amino acid-to-tyrosine ratio (BTR) is a prognostic predictive factor in patients with liver cirrhosis by determining the relationship of the BTR with event-free survival in a retrospective, observational cohort study. The medical records of patients with liver cirrhosis who visited our institution from February 2000 to May 2012 were examined. Events due to liver cirrhosis were defined as death, worsening of esophageal and/or gastric varices, hepatocellular carcinoma, and liver failure. The primary endpoint was the period from the date of BTR measurement until the first onset of these events. Event-free survival was compared between patients with BTR ≥ 4 and BTR < 4. Relationships between the BTR and other factors predicting prognosis were also examined. Event-free survival was evaluated in patients with and without branched-chain amino acid supplementation using propensity score matching. Significantly longer event-free survival was found in liver cirrhosis patients with BTR ≥ 4 (*n =* 425) compared with those with BTR < 4 (*n =* 105), and the BTR was associated with liver cirrhosis events. The BTR showed significant relationships with other predictive factors evaluated. In subcohorts matched by propensity score, branched-chain amino acid supplementation significantly improved event-free survival in patients with BTR <4. The BTR is clinically useful for predicting prognosis in liver cirrhosis patients. BCAA supplementation may be beneficial in those with BTR < 4.

## INTRODUCTION

Liver cirrhosis is an advanced stage of chronic liver disease and a public health issue due to its high mortality rate [1]. Hepatitis virus infection (type B and type C) is the leading etiological cause of liver cirrhosis, followed by chronic alcohol consumption. Recently, the number of patients with nonalcoholic fatty liver disease (NAFLD)- or nonalcoholic steatohepatitis (NASH)-associated liver cirrhosis has increased due to metabolic dysfunction with type 2 diabetes mellitus and dyslipidemia [2]. Hepatocyte injury due to these causes induces and enhances inflammatory necrosis or fibrosis, leading to liver cirrhosis [3, 4].

Since the liver is the major organ metabolizing fat, protein, and carbohydrates, protein-energy malnutrition and hepatic malnutrition due to dysfunction of hepatocytes is frequently noted among patients with liver cirrhosis. In this case, decreased albumin levels are due to the reduced ability to synthesize protein [5]. In patients with liver cirrhosis, increased enzyme levels leaked from hepatocytes, decreased platelet counts, prolonged prothrombin times, increased bilirubin levels due to cholestasis, etc. are clinically complicated signs and symptoms. Several indicators were proposed to estimate not only the severity of liver cirrhosis and progression of hepatic fibrosis but also the prognosis of cirrhosis based on the values from laboratory examinations and/or symptoms. Currently, the Child-Pugh score [6], FIB-4 index [7], albumin-bilirubin (ALBI) grade [8], method of end-stage liver disease (MELD) score [9], and MELD-Na score [10] are utilized clinically as predictive factors of the severity, fibrosis progression, and prognosis of liver cirrhosis patients.

Imbalanced amino acid metabolism is induced by the consumption of the essential branched-chain amino acids (BCAAs; valine, leucine, and isoleucine), which are energy substrates in muscles. Patients with liver cirrhosis therefore have a lower concentration of BCAA but a higher level of aromatic amino acids (AAAs) [11, 12]. As an evaluable indicator of energy metabolism, the BCAA-to-AAA ratio or BCAA-to-tyrosine ratio (BTR) is utilized [13]. The BTR is referred to as a decision marker of therapeutic direction for patients with liver cirrhosis in our institution, and we reported that it was a predictive factor for early-phase hepatocellular carcinoma. The survival rate in patients with a high BTR (≥ 4) is significantly greater than that in patients with a low BTR (< 4) [14].

We evaluated whether the BTR is a predictive indicator of prognosis in patients with liver cirrhosis to determine the relationship of the BTR with survival and worsening disease (occurrence of complications) in the clinical setting in a retrospective, observational cohort study. The clinical significance of the BTR was also investigated to compare it with other predictive indicators. In this study, the Child-Pugh score, FIB-4 index, ALBI grade MELD score, and MELD-Na score were employed as comparative indicators. In addition, an investigation of the effects of BCAA supplementation therapy on prognosis in patients with liver cirrhosis was conducted as an exploratory evaluation using propensity score matching [15].

## RESULTS

### Patient characteristics

**Figure 1:**
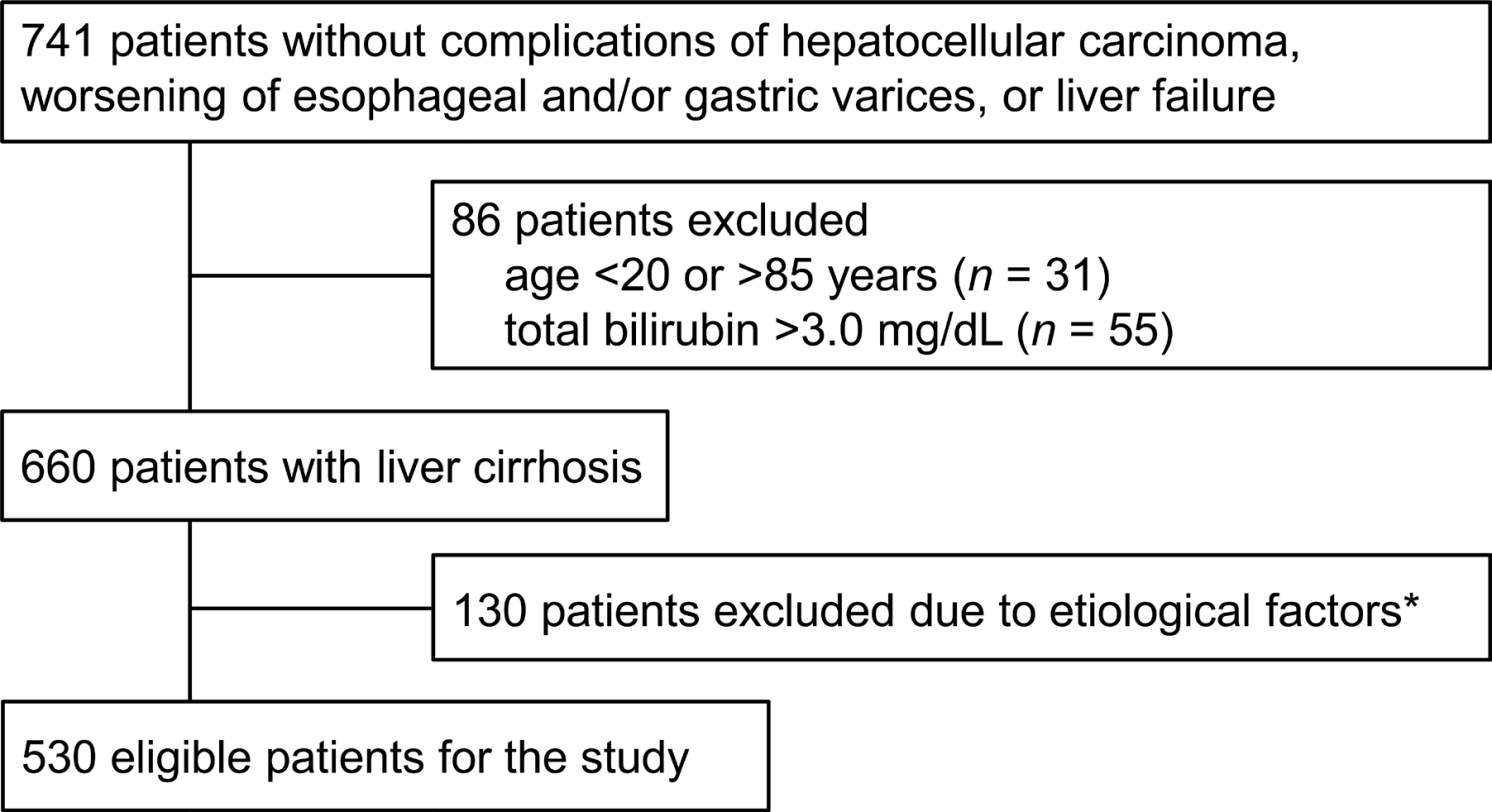
Flow-chart of the patient data utilized in the study *The etiological factors of liver cirrhosis were limited to hepatitis C and hepatitis B virus infection, autoimmune hepatitis, primary biliary cirrhosis, idiopathic portal hypertension, NASH, or alcoholic hepatitis.

Among 1,223 patients with liver cirrhosis who first visited Saiseikai Niigata Daini Hospital from February 2000 to May 2012, 741 met the selection criteria. As shown in Figure 1, 530 patients were finally selected for the study analysis in accordance with the other selection criteria of age, total bilirubin level, and etiology of liver cirrhosis. Baseline patient characteristics are shown in Table 1.

**Table 1:** Patient characteristics

	Total (*N* = 530)		high BTR group (*N* = 425)		Low BTR group (*n =* 105)			
	*n* (%) or mean ± SD		*n* (%) or mean ± SD		*n* (%) or mean ± SD		*p* value	
Gender (male)	299	(56.4)	252	(59.3)	47	(44.8)	0.0083	(F)
Age (years)	62.5 ± 13.1		61.2 ± 13.2		68.0 ± 11.1		< 0.0001	(W)
Platelet (×10^4^/μL)	17.0 ± 7.1		18.1 ± 6.8		12.5 ± 6.5		< 0.0001	(W)
Prothrombin time (sec)	11.79 ± 1.39		11.53 ± 1.14		12.70 ± 1.77		< 0.0001	(W)
Total bilirubin (mg/dL)	0.80 ± 0.50		0.74 ± 0.46		1.02 ± 0.57		< 0.0001	(W)
AST (U/L)	78.6 ± 144.8		75.9 ± 156.2		89.4 ± 85.8		< 0.0001	(W)
ALT (U/L)	92.0 ± 185.8		93.0 ± 202.7		88.0 ± 92.6		0.0741	(W)
Albumin (mg/dL)	3.90 ± 0.56		3.97 ± 0.51		3.62 ± 0.68		< 0.0001	(W)
BTR	5.66 ± 2.07		6.27 ± 1.84		3.21 ± 0.64		< 0.0001	(W)
Child-Pugh score	5.7 ± 1.3		5.4 ± 0.8		6.7 ± 2.0		< 0.0001	(W)
FIB-4 index	4.1 ± 4.1		3.4 ± 3.8		6.8 ± 4.0		< 0.0001	(W)
ALBI grade	–2.6 ± 0.5		–2.7 ± 0.4		–2.3 ± 0.7		< 0.0001	(W)
MELD score	8.2 ± 2.6		7.8 ± 2.5		9.4 ± 2.8		< 0.0001	(W)
MELD-Na score	9.2 ± 3.3		8.8 ± 3.1		10.6 ± 3.8		< 0.0001	(W)
Etiological factor							0.0003	(C)
HCV	245	(46.2)	188	(44.2)	57	(54.3)		
HBV	80	(15.1)	70	(16.5)	10	(9.5)		
AIH	24	(4.5)	13	(3.1)	11	(10.5)		
PBC	27	(5.1)	24	(5.6)	3	(2.9)		
IPH	0	(0.0)	0	(0.0)	0	(0.0)		
NASH	52	(9.8)	50	(11.8)	2	(1.9)		
AH	88	(16.6)	70	(16.5)	18	(17.1)		
Multiple factors	14	(2.6)	10	(2.4)	4	(3.8)		

*BTR* branched-chain amino acid-to-tyrosine ratio, *FIB-4* fibrosis 4, *ALBI* albumin-bilirubin, *MELD* model for end-stage liver disease, *MELD-Na* MELD sodium, *HVC* hepatitis C, *HVB* hepatitis B, *AIH* autoimmune hepatitis, *PBC* primary biliary cirrhosis, *IPH* idiopathic portal hypertension, *NASH* nonalcoholic steatohepatitis, *AH* alcoholic hepatitis. *F* high BTR group: BTR ≥ 4, low BTR group: BTR < 4. Fisher’s exact test, *W* Wilcoxon’s test, C *χ*^2^ test. *p* value: comparison between high BTR and low BTR groups

Overall, the ratio of men (56.4%) was slightly high, and the mean age was 62.5 years. The mean BTR was 5.66 ± 2.07, and 425 (80.2%) patients had BTR ≥ 4 at baseline (high BTR group). Patients with BTR < 4 (*n* = 105, low BTR group) were older and had more severe cirrhosis based on parameters excluding the ALT level. There was no difference in the treatment of liver cirrhosis between the two groups. The mean observation period was 62 months, and the median was 54 months (0 to 169 months).

### Relationship between the BTR and all events due to liver cirrhosis

Table 2 shows all events and individual events in both groups. Forty-three (8.1%) patients died, and worsening of esophageal and/or gastric varices occurred in 62 (11.7%), hepatocellular carcinoma in 54 (10.2%), and liver failure in 26 (4.9%). Overall, events occurred in 106 patients (20.0%). The incidence of these events was higher in the low BTR group (53.3%) than that in the high BTR group (11.8%).

**Table 2:** Events due to liver cirrhosis

	All cases (*n* = 530)		High BTR group (*n* = 425)		Low BTR group (*n* = 105)	
All events	106	(20.0)	50	(11.8)	56	(53.3)
Death	43	(8.1)	20	(4.7)	23	(21.9)
Worsening of esophageal and/or gastric varices	62	(11.7)	22	(5.2)	40	(38.1)
Hepatocellular carcinoma	54	(10.2)	22	(5.2)	32	(30.5)
Liver failure	26	(4.9)	6	(1.4)	20	(19.0)
Worsening of esophageal and/or gastric varices + liver failure	69	(13.0)	25	(5.9)	44	(41.9)

*BTR* branched-chain amino acid-to-tyrosine ratio, *high BTR group* BTR ≥ 4, *low BTR group* BTR < 4.

**Figure 2:**
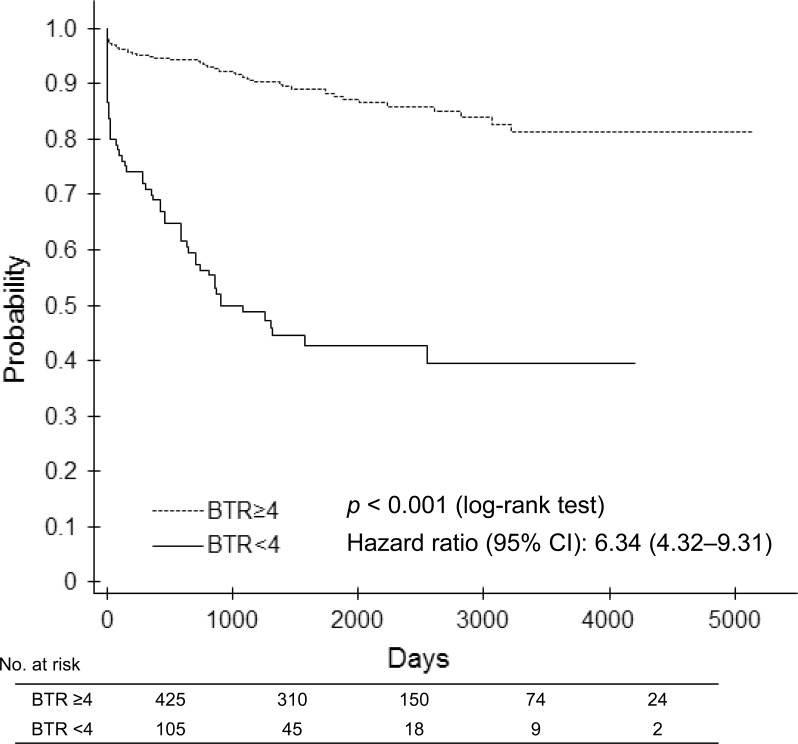
Event-free survival curve between the low (< 4) BTR and high (≥ 4) BTR groups

Event-free survival curves generated by the Kaplan-Meier method for each group are shown in Figure 2, and the hazard ratio of all events was 6.34 (95% confidence interval [CI] 4.32 to 9.31; *p* < 0.0001] between the two groups. The hazard ratio of death, worsening of esophageal and/or gastric varices + liver failure, and hepatocellular carcinoma were 4.50 (95% CI: 2.47 to 8.20; *p* < 0.0001), 9.09 (95% CI: 5.55 to 14.87; *p* < 0.0001), and 6.63 (95% CI: 3.84 to 11.42; *p* < 0.0001), respectively.

### Correlation between the BTR and other predictive factors

**Figure 3:**
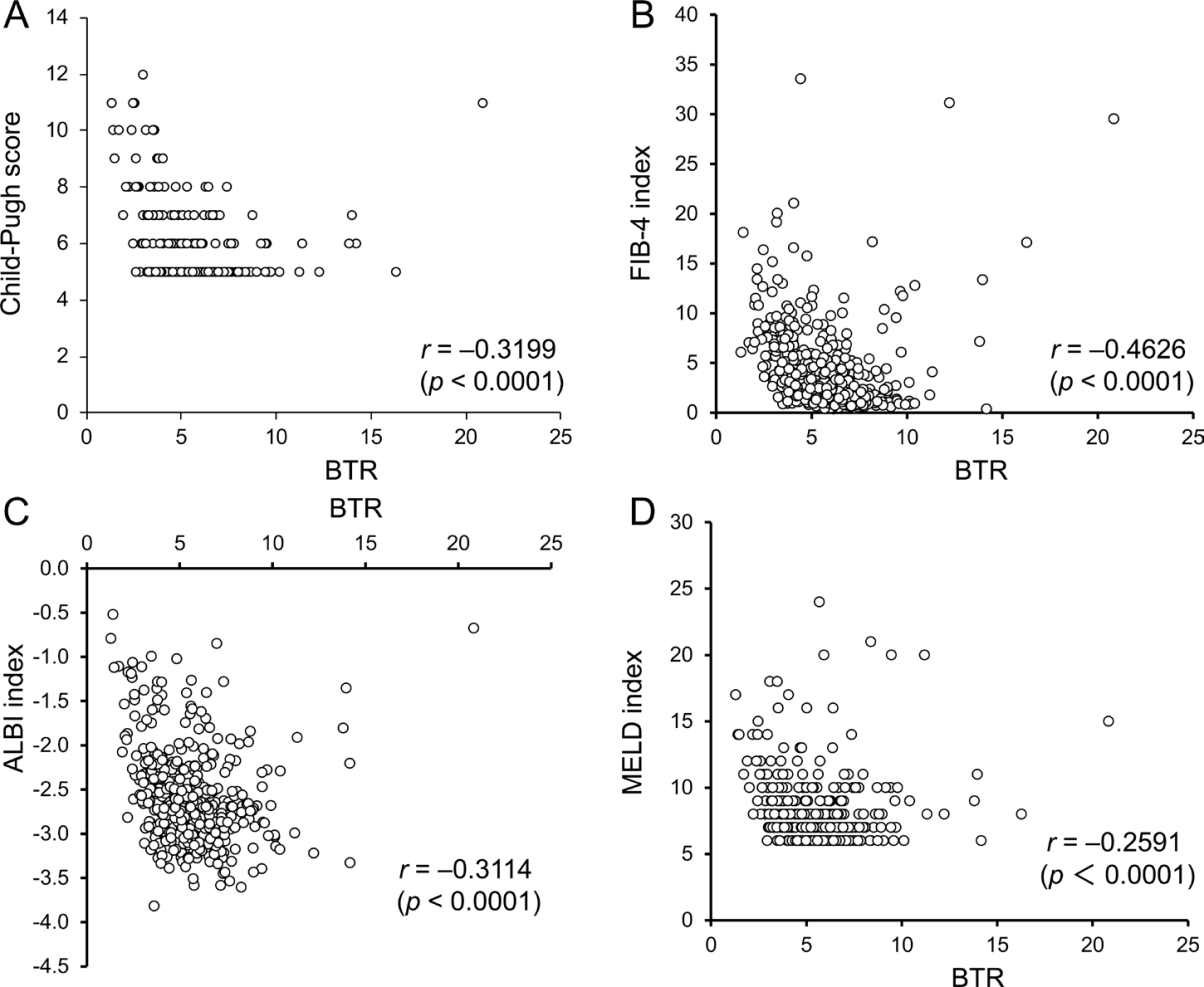

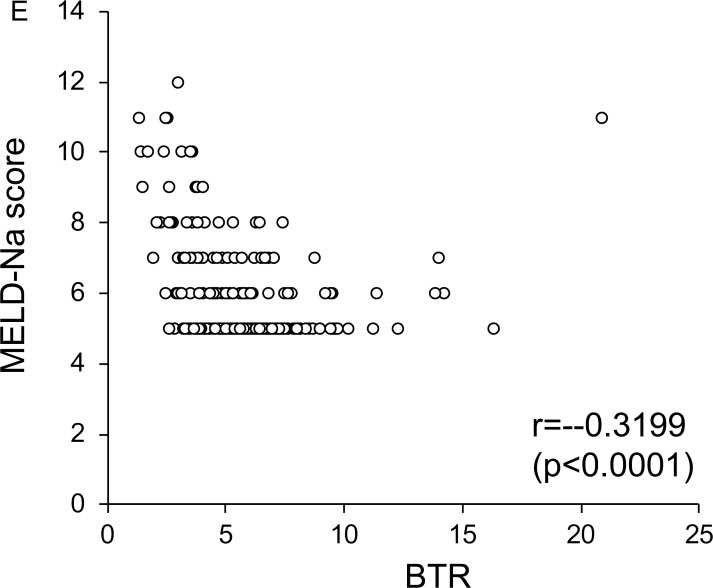
Relationship of the BTR with the Child-Pugh score (**A**), FIB-4 index (**B**), ALBI grade (**C**), MELD score (**D**), and MELD-Na score (**E**).

All six predictive factors showed a significant correlation with the BTR value: *r* = 0.3199 (*p* < 0.0001) for the Child-Pugh score; *r* = 0.426 (*p* < 0.0001) for the FIB-4 index; *r* = 0.3114 (*p* < 0.0001) for the ALBI grade; *r* = 0.2591 (*p* < 0.0001) for the MELD score; and *r* = 0.1401 (*p* = 0.0058) for the MELD-Na score. However, the correlations were not strong (Figure 3).

### Relationship between other predictive factors and all events

**Figure 4:**
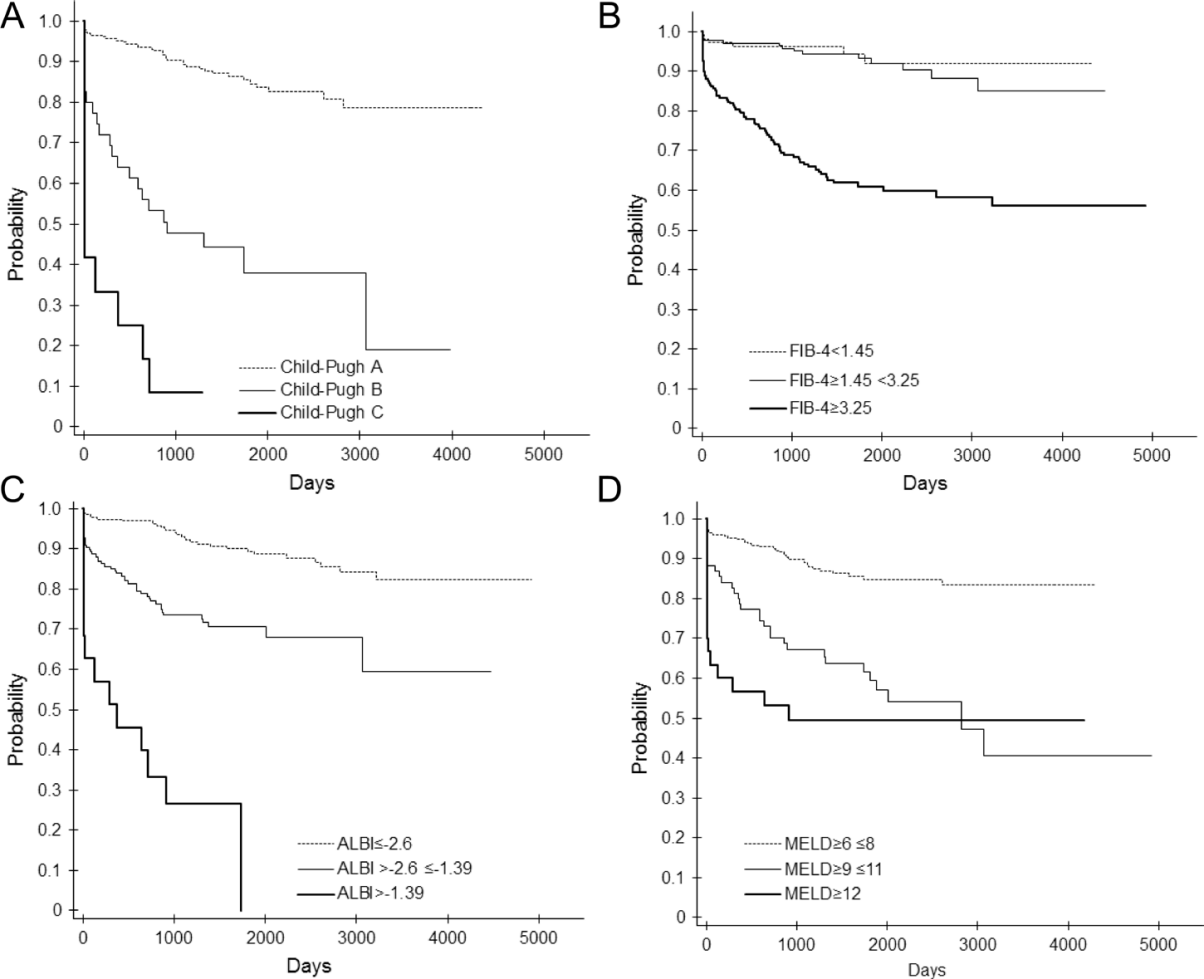

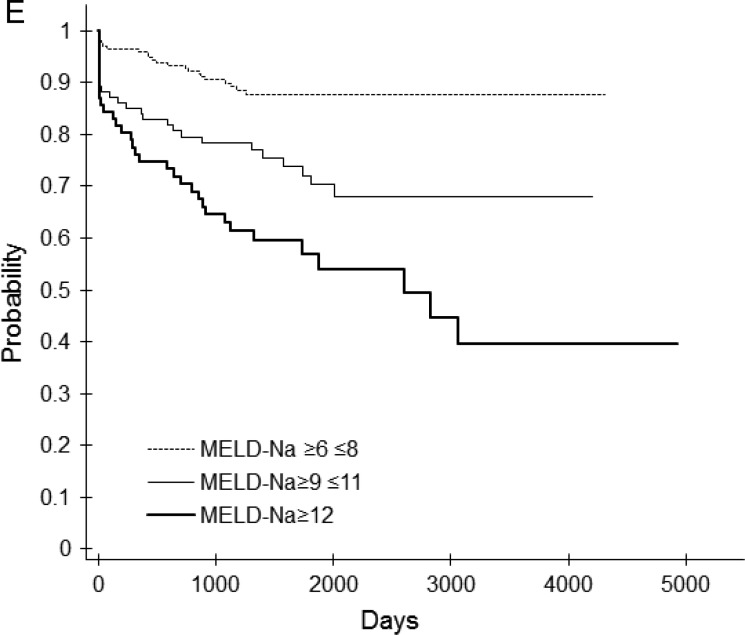
Event-free curve according to the Child-Pugh score (**A**), FIB-4 index (**B**), ALBI grade (**C**), MELD score (**D**), and MELD-Na score (**E**).

The Kaplan-Meier curves for event-free survival with the categorized severity of each predictive factor (Child-Pugh score, FIB-4 index, ALBI grade, MELD score, or MELD-Na score) are shown in Figure 4. The severity category of each factor was significantly associated with event-free survival. The hazard ratio for the severity category of each factor is shown in Table 3.

**Table 3:** Comparisons of hazard ratios of all events for each prognostic predictive factor for liver cirrhosis

	*n*	No. of events	(%)	Hazard ratio*	95 % CI	*p* value
Child-Pugh grade						
A	260	36	(13.8)	1.00		
B	41	23	(56.1)	6.14	3.62–10.42	< 0.0001
C	12	11	(91.7)	23.04	11.31–46.94	< 0.0001
FIB-4 index						
< 3.25	290	20	(6.9)	1.00		
≥ 3.25	219	80	(36.5)	6.13	3.76–10.01	< 0.0001
ALBI grade						
≤ –2.6	277	30	(10.8)	1.00		
> –2.6 ≤ –1.39	180	49	(27.2)	3.45	2.17–5.48	< 0.0001
> –1.39	19	14	(73.7)	17.04	8.84–32.85	< 0.0001
MELD score						
6–8	282	36	(12.8)	1.00		
9–	106	47	(44.3)	4.08	2.65–6.31	< 0.0001
MELD-Na score						
6–8	206	22	(10.7)	1.00		
9–11	103	27	(26.2)	2.72	1.55–4.78	0.0005
12–	77	34	(44.2)	4.91	2.87–8.40	< 0.0001

*FIB-4* fibrosis 4, *ALBI* albumin-bilirubin, *MELD* model for end-stage liver disease, *MELD-Na* MELD sodium, *CI* confidential interval. *Cox proportional hazard model was utilized.

### Multivariate analysis of the prediction of event occurrence

Multivariate analysis revealed that the BTR, Child-Pugh score, Fib-4 index, and gender (female) were significantly strong contributing factors to the occurrence of all composite events (Table 4). Among independent variables, however, age was not a contributing factor.

**Table 4:** Multivariate analysis for all composite events

Factor	Hazard ratio*	95 % CI	*p* value
BTR index	0.77	0.67–0.89	0.0002
Child-Pugh score	1.55	1.31–1.82	< 0.0001
Fib-4 index	1.10	1.05–1.15	< 0.0001
Gender (female)	0.43	0.25–0.75	0.0030

Independent factors: BTR, Fib-4 index, ALBI index, MELD Na index, Child-Pugh score, gender, age, and etiological factor. *BTR* branched-chain amino acid-to-tyrosine ratio, *FIB-4* fibrosis 4, *CI* confidence interval. *Cox proportional hazards model was utilized.

### Evaluation of BCAA supplementation therapy in patients matched by propensity score

In both the low and high BTR groups, propensity score matching was performed between patients administered BCAA supplementation therapy (BCAA^+^ group) and corresponding patients who were not (BCAA^–^ group). Patient characteristics at baseline were similar between the BCAA^–^ and BCAA^+^ groups, as shown in Table 5.

**Table 5:** Patient characteristics matched with propensity score

	BTR < 4						BTR ≥ 4					
	BCAA^–^ *n =* 15		BCAA^+^ *n =* 15				BCAA^–^ *n =* 18		BCAA^+^ *n =* 18			
	*n* (%) or mean ± SD		*n* (%) or mean ± SD		*p value*		*n* (%) or mean ± SD		*n* (%) or mean ± SD		*p* value	
Gender (male)	8	(53.3)	7	(46.7)	1.0000	(F)	7	(38.9)	7	(38.9)	1.0000	(F)
Age (years)	64.5 ± 10.8		67.3 ± 13.6		0.3720	(W)	64.9 ± 12.5		64.9 ± 11.1		0.9495	(W)
Interferon (yes)	6	(40.0)	7	(46.7)	1.0000	(F)	9	(50.0)	8	(44.4)	1.0000	(F)
Nucleic acid analogue formulation (yes)	2	(13.3)	2	(13.3)	1.0000	(F)	1	(5.6)	2	(11.1)	1.0000	(F)
Platelet (×10^4^/μL)	11.7 ± 8.0		10.6 ± 4.0		0.6333	(W)	18.8 ± 6.5		19.9 ± 10.3		0.9118	(W)
Prothrombin time (%)	13.06 ± 1.50		13.63 ± 2.93		0.5335	(W)	11.49 ± 1.46		11.59 ± 1.13		0.5578	(W)
Total bilirubin (mg/dL)	1.30 ± 0.67		1.25 ± 0.74		0.5069	(W)	0.69 ± 0.27		0.64 ± 0.33		0.2961	(W)
AST (U/L)	77.5 ± 62.5		89.5 ± 58.4		0.6631	(W)	64.1 ± 76.4		58.1 ± 47.7		0.6237	(W)
ALT (U/L)	60.5 ± 61.9		73.1 ± 60.1		0.4936	(W)	96.2 ± 155.5		49.6 ± 45.3		0.1833	(W)
Albumin (mg/dL)	3.2 ± 0.7		3.3 ± 0.8		0.5744	(W)	4.0 ± 0.4		3.8 ± 0.4		0.3396	(W)
BTR	3.03 ± 0.65		3.14 ± 0.76		0.4935	(W)	6.40 ± 1.62		6.57 ± 2.03		0.9244	(W)
Child-Pugh score	7.7 ± 2.6		7.1 ± 1.9		0.6270	(W)	5.3 ± 0.7		5.3 ± 0.5		0.6276	(W)
FIB-4 index	8.3 ± 5.9		7.4 ± 3.6		0.9504	(W)	3.0 ± 1.9		3.4 ± 2.7		0.7758	(W)
ALBI grade	–1.8 ± 0.7		–2.0 ± 0.7		0.6334	(W)	–2.7 ± 0.3		–2.6 ± 0.3		0.6464	(W)
MELD score	10.9 ± 4.0		9.7 ± 3.0		0.4393	(W)	7.3 ± 1.4		7.2 ± 1.5		0.7906	(W)
MELD-Na score	12.2 ± 4.9		10.9 ± 4.1		0.5192	(W)	8.3 ± 2.2		8.2 ± 2.3		0.8343	(W)

*BTR* BCAA-to-tyrosine ratio, *BCAA* branched-chain amino acids, *BCAA*^*–*^ treatment without BCAA, *BCAA*^*+*^ treatment with BCAA, *FIB-4* fibrosis 4, *ALBI* albumin-bilirubin, *MELD* model for end-stage liver disease, *MELD-Na* MELD sodium, *F* Fisher’s exact test, *W* Wilcoxon’s test.

**Figure 5:**
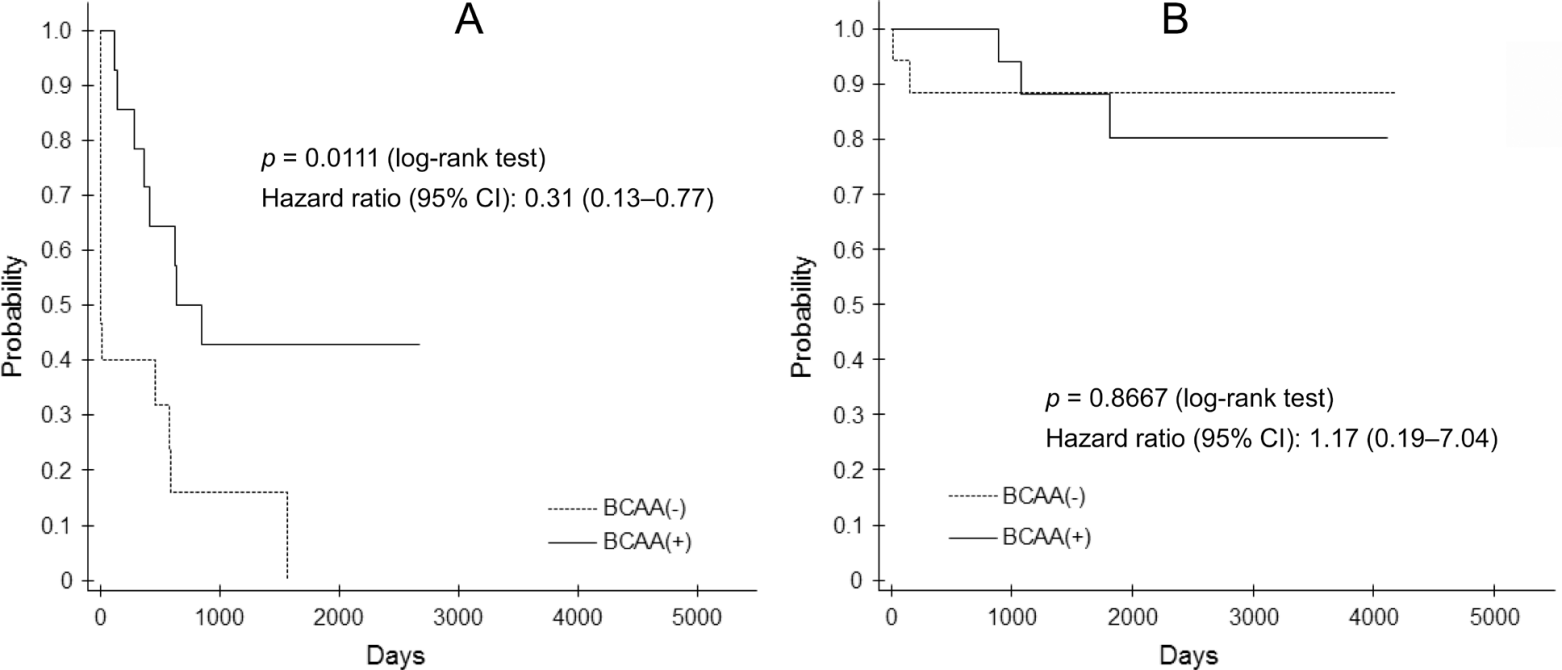
Event-free survival curve between patients with BTR < 4 (**A**) and ≥ 4 (**B**) in the BCAA^–^ and BCAA^+^ groups.

Event-free survival curves according to the BTR subgroup in the BCAA^–^ and BCAA^+^ groups are shown in Figure 5. In patients with BTR < 4, BCAA supplementation therapy significantly improved the event-free survival rate with a hazard ratio of 0.31 (95% CI: 0.13 to 0.77; *p* = 0.0111), although it did not in patients with BTR ≥ 4.

## DISCUSSION

In this retrospective, observational study to evaluate event-free survival in patients with liver cirrhosis, the BTR was a clinically useful factor for the prediction of long-term progression. Event-free survival in patients with BTR ≥ 4 was significantly longer than that in those with BTR < 4, and there was a significant correlation between the BTR and other predictive factors. In addition, BCAA supplementation improved the prognosis of patients with BTR < 4.

The complications of liver cirrhosis which is the end stage of advanced chronic liver disease often result in death as well as deterioration in the activities of daily living and quality of life, and therefore improving the prognosis of liver cirrhosis is a clinically important issue [16]. Since the process of liver fibrosis is reversible, not only aggressive treatment of fibrosis but also appropriate therapy for the underlying etiology of cirrhosis may improve patient prognosis. It is therefore important to assess both the severity of liver cirrhosis and degree of fibrosis.

Several disease severity evaluations based on values from laboratory tests are clinically utilized due to their low cost and repeatability. The Child-Pugh score is based on clinical symptoms (ascites and encephalopathy), bilirubin and albumin levels, and prothrombin time [6]. The MELD score is calculated from creatinine and bilirubin levels and the International Normalized Ratio of prothrombin time [9]. It was reported that the Child-Pugh and MELD scores are good predictors of the prognosis of liver cirrhosis patients in the clinical setting, although the MELD score is superior to the Child-Pugh score because it does not involve clinical symptoms [17]. Since the sodium level itself is an independent predictive factor of liver cirrhosis prognosis, the MELD-Na score including the sodium level is also utilized [18]. The ALBI grade calculated from albumin and bilirubin levels is considered to be a prognosis predictor like the Child-Pugh score in patients with hepatocellular carcinoma [19]. The FIB-4 index is scored based on age, AST and ALT levels, and platelet count [7] and was also reported to be a prognosis predictor [20]. The present study also found that these indexes predicted the prognosis of liver cirrhosis.

As described above, theses indexes consist of multiple factors. This study mainly evaluated whether the BTR could be employed to predict prognosis, since protein-energy malnutrition is induced by lower metabolism of amino acids in advanced liver cirrhosis. Also, BTR is considered a simpler index than other indexes, since BTR can be calculated by only amino acids ratio by one time measurement of blood sample Event-free survival in liver cirrhosis patients with BTR ≥ 4 was significantly longer than that in patients with BTR < 4, when the cut-off value in the BTR was set at 4 as in a previous report [14]. Since the disease status of liver cirrhosis at the cut-off of each index may not be the same, direct comparison of the Kaplan-Meier curves is not appropriate. Accordingly, no comparison of correlations between the BTR and other predictive factors was performed. Significant, but not strong, correlations were found, however. It is considered that this phenomenon may be due to heterogeneous phenotypes of liver cirrhosis. Although the BTR was evaluated as a predictive index for patients with hepatocellular carcinoma [14, 15], this study revealed that the BTR may be effective index for predicting the occurrence of complications in patients with liver cirrhosis which is a pre-stage of hepatocellular carcinoma.

The multivariate analysis of all composite events as a dependent variable in the study patients indicated that the BTR, Child-Pugh score, and FIB-4 index as well as gender (female) were significant factors affecting prognosis. This indicates that multiple predictive factors should be employed for the estimation of prognosis in patients with liver cirrhosis, because multiple promoting factors are associated with prognosis.

Aggressive therapy to prevent the worsening of liver cirrhosis and fibrosis is required. Since the BTR is a factor predicting prognosis, BCAA supplementation therapy was administered to cirrhosis patients to improve their serum albumin concentration. A 2-year observational study was performed to evaluate event-free survival in patients with liver cirrhosis who received oral BCAA 12 g in addition to their usual nutritional regimen [21]. Patients with serum albumin < 3.5 g/dL who received BCAA supplementation showed a significantly higher event-free survival rate than those who did not (hazard ratio = 0.67; *p* = 0.015). In a meta-analysis of BCAA supplementation therapy, increases in albumin levels and reductions in ascites and edema were reported. BCAA supplementation did not improve the mortality rate at the 1-year evaluation, although at the 3-year observation the mortality rate was significantly decreased [22]. It may, however, be necessary to consider the condition and status of patents with liver cirrhosis when selecting for BCAA supplementation therapy to achieve the optimum effects.

We evaluated the beneficial effects of BCAA supplementation in patients with liver cirrhosis using propensity score matching as an exploratory study. In patients with BTR ≥ 4, BCAA supplementation did not reduce the occurrence of all composite events, although it improved prognosis significantly in patients with BTR < 4. Taking the results together, it will be necessary to define the patient group to receive BCAA supplementation therapy. However, our propensity score matching results come only from a retrospective, small-group comparison. A large-scale, prospective clinical study will be required to clarify which liver cirrhosis patients should be administered BCAA supplementation.

## MATERIALS AND METHODS

### Study design

This study was a single-center, retrospective, observational cohort study to evaluate the clinical significance of the BTR as a predictive factor in patients with liver cirrhosis. Medical records and information from Saiseikai Niigata Daini Hospital were analyzed. Since this was a retrospective study using existing medical records and information, informed consent was not required. However, study patients were given the opportunity to refuse the release of their medical records and information for analysis after study approval had been obtained from the Medical Ethical Committee of Saiseikai Niigata Daini Hospital.

### Study patients

The medical records and information of sequential patients who were first diagnosed with liver cirrhosis at Saiseikai Niigata Daini Hospital from February 2000 to May 2012 were analyzed. The inclusion criteria for extracting data were: 1) the BTR value was measured at the first visit; 2) BCAA supplementation was not administered prior to BTR measurement; 3) the final outcome was assessable; 4) there were no complications of hepatocellular carcinoma, worsening of esophageal and/or gastric varices or liver failure (ascites, edema, or hepatic encephalopathy); and 5) patients were aged from 20 to 85 years at the first visit. Patients whose total bilirubin level was ≥ 3.0 mg/dL were excluded. The etiological factors of liver cirrhosis were limited to hepatitis C and hepatitis B virus infection, autoimmune hepatitis, primary biliary cirrhosis, idiopathic portal hypertension, NASH, or alcoholic hepatitis, with other etiological factors excluded.

### Parameters evaluated

The first date of BTR measurement was defined as baseline, and the period until the first occurrence of liver cirrhosis-related events (death, worsening of esophageal and/or gastric varices, hepatocellular carcinoma, and liver failure) from the date of BTR measurement was determined. As the primary endpoint, event-free survival was compared between patients with BTR < 4 (low BTR group) and with BTR ≥ 4 (high BTR group). The secondary endpoint was the event-free survival rate in the two groups. The Child-Pugh score, FIB-4 index, ALBI grade, MELD score, and MELD-Na index were employed as comparable factors since they are clinically utilized as predictors of severity and fibrosis in liver cirrhosis. To investigate the clinical significance of the BTR for predicting prognosis in patients with liver cirrhosis, a single correlation of the BTR with the predictive factors above was evaluated. Since the normal range of the BTR is reported as 4.41–10.05, the cut-off value was employed as 4 in this study.

In addition, patients who were treated with and without BCAA supplementation were selected among patients analyzed in this study by propensity score matching to investigate event-free survival based on the BTR value (< 4 or ≥ 4) at baseline in matched patients as an exploratory evaluation. For propensity matching, gender, age, BTR, Child-Pugh score, FIB-4 index, ALBI grade, MELD score, and MELD-Na score were used as covariates. An oral nutritional supplement for liver failure or BCAA preparation was administered.

### Statistics

The medical records and information were made anonymous prior to analysis to prevent individual patients from being identified. Descriptive data are presented as the mean ± standard deviation for continuous variables and as counts and percentages for categorical variables, unless otherwise specified. Sample size estimation was not performed due to the retrospective observational analysis of existing medical records and information. Event-free survival rates in the two groups based on the BTR value (< 4 or ≥ 4) were compared using the Kaplan-Meier method and log-rank test. Hazard ratios of all composite events between the two groups were determined with the Cox proportional hazards model. For the six predictive factors, the Kaplan-Meier method and Cox proportional hazards model were similarly performed for each. Multivariate analysis of all composite events as a dependent variable was performed with the BTR, Child-Pugh score, FIB-4 index, ALBI grade MELD Na score, gender, age, and etiological factors as independent variables. Relative frequencies of background parameters were compared using Fisher’s exact test, Wilcoxon rank-sum test, and χ2 test. Statistical analysis was performed at Satt Co., Ltd. (Tokyo, Japan) using SAS ver.9.4 (SAS Institute, Cary, NC, USA) with the two-sided test. A *p* value of less than 0.05 was considered to represent a statistically significant difference.

## CONCLUSIONS

It is important to assess the severity of prognosis in patients with liver cirrhosis. The BTR is a convenient factor to estimate prognosis in the clinical setting and it is a useful parameter for judging the timing of interventional treatment with BCAA supplementation in liver cirrhosis.
